# Natural killer cell responses during SARS-CoV-2 infection and vaccination in people living with HIV-1

**DOI:** 10.1038/s41598-023-45412-9

**Published:** 2023-11-03

**Authors:** Aljawharah Alrubayyi, Emma Touizer, Dan Hameiri-Bowen, Bethany Charlton, Ester Gea-Mallorquí, Noshin Hussain, Kelly A. S. da Costa, Rosemarie Ford, Chloe Rees-Spear, Thomas A. Fox, Ian Williams, Laura Waters, Tristan J. Barber, Fiona Burns, Sabine Kinloch, Emma Morris, Sarah Rowland-Jones, Laura E. McCoy, Dimitra Peppa

**Affiliations:** 1 Nuffield Department of Medicine, University of Oxford, Oxford, UK; 2 Division of Infection and Immunity, Institute for Immunity and Transplantation, University College London, London, UK; 3 Department of HIV, Mortimer Market Centre, Central and North West London NHS Trust, London, UK; 4 Institute for Global Health, University College London, London, UK; 5 The Ian Charleson Day Centre, Royal Free Hospital NHS Foundation Trust, London, UK

**Keywords:** Virology, HIV infections, Viral infection, Immunology

## Abstract

Natural killer (NK) cell subsets with adaptive properties are emerging as regulators of vaccine-induced T and B cell responses and are specialized towards antibody-dependent functions contributing to SARS-CoV-2 control. Although HIV-1 infection is known to affect the NK cell pool, the additional impact of SARS-CoV-2 infection and/or vaccination on NK cell responses in people living with HIV (PLWH) has remained unexplored. Our data show that SARS-CoV-2 infection skews NK cells towards a more differentiated/adaptive CD57^+^FcεRIγ^−^ phenotype in PLWH. A similar subset was induced following vaccination in SARS-CoV-2 naïve PLWH in addition to a CD56^bright^ population with cytotoxic potential. Antibody-dependent NK cell function showed robust and durable responses to Spike up to 148 days post-infection, with responses enriched in adaptive NK cells. NK cell responses were further boosted by the first vaccine dose in SARS-CoV-2 exposed individuals and peaked after the second dose in SARS-CoV-2 naïve PLWH. The presence of adaptive NK cells associated with the magnitude of cellular and humoral responses. These data suggest that features of adaptive NK cells can be effectively engaged to complement and boost vaccine-induced adaptive immunity in potentially more vulnerable groups such as PLWH.

## Introduction

With an evolving pandemic and the emergence of new variants, SARS-CoV-2 infection continues to be a global health concern. Despite the great strides in vaccine development against SARS-CoV-2, there are remaining gaps in our understanding of how to elicit broadly protective and durable immunity, especially in potentially vulnerable groups. People living with HIV-1 (PLWH) are thought to be at increased risk of worse disease outcomes, especially in the context of advanced immunodeficiency, co-morbidities and/or unsuppressed HIV-1 viremia^1–3^. As a result, in the UK, PLWH were prioritized for COVID-19 vaccination and additional vaccine doses^4^. Emerging evidence supports that some PLWH, despite effective antiretroviral therapy (ART), generate a poorer neutralizing antibody response to vaccination reflective of defects in the B cell compartment that could increase the risk for SARS-CoV-2 reinfection^5–8^. Whereas adaptive immune responses have been more extensively characterized in this patient group, the role of innate effectors such as NK cells that can play a role in viral control and shaping adaptive immunity is less well understood^9–11^.

NK cells provide early host defense against viral infections^12^. They can directly eliminate virus-infected cells and produce cytokines and chemokines that can mediate antiviral effects and stimulate robust adaptive responses, facilitating viral control^12^. In addition to their innate activity, they can contribute to the durability of vaccine responses, including memory responses (reviewed in^13^). In COVID-19 disease, some studies have shown an association between specific NK cell pro-inflammatory phenotypes and disease severity^14–20^. Notably, NK cell depletion in peripheral blood, exhaustion, and impaired function during acute SARS-CoV-2 infection have been linked to worse disease outcomes, indicating that SARS-CoV-2 infection can affect NK cell redistribution and effector function^14–16^. The contribution of NK cells to SARS-CoV-2 immunity is supported by studies showing that NK cell-mediated Fc-dependent functions and antibody-dependent cellular cytotoxicity (ADCC) can provide functional antiviral activity and suppress viral replication^18,19,21–24^. Anti-SARS-CoV-2 antibodies, not restricted to the Spike protein, have been shown to induce NK cell-mediated ADCC responses following natural infection^23,24^. Similarly, SARS-CoV-2 vaccination mediated strong NK cell activation in HIV-negative vaccine recipients^22,25^. Polyfunctional antibody responses with the capacity to mediate strong ADCC responses have been linked to favorable COVID-19 disease outcomes^26,27^ and vaccine protection in non-human primates^28–30^. These ADCC responses decay slowly after natural infection or vaccination, persisting beyond neutralizing antibody responses and could, therefore, be important for long-term immunity^25,27,31,32^. Importantly, Fc effector functions are preserved against neutralization-resistant SARS-CoV-2 variants of concern (VOCs), highlighting the potential supportive role of other immune functions in protection against severe disease^27,33,34^. This could be pertinent to PLWH, in whom neutralizing responses to the ancestral strain and SARS-CoV-2 VOCs have been shown to be reduced^5–7,35^.

Our group and others have previously described an adaptive reconfiguration of NK cells in HIV-1 infection with preserved ADCC capacity^36–39^. These populations are partly driven by human cytomegalovirus (HCMV) co-infection, which is highly prevalent in PLWH, and can be further induced by pro-inflammatory cytokines^37,40–43^. The complex interplay between HCMV and NK cells can leave a long-lasting imprint on the NK cell compartment and affect responses toward new pathogens and vaccines^44,45^. These adaptive NK cells are characterized by higher expression of NKG2C, an activating receptor which recognizes HLA-E, and loss of expression of the proximal signaling molecule, FcεRI-γ^40,41,46–48^. They have a more selective recognition repertoire (oligoclonal pattern of Killer Immunoglobulin-like receptors (KIRs)) and are functionally distinct with a strong Fc receptor-dependent effector function^36,40,41,46,47,49^. Adaptive NK cells share epigenetic similarities with memory CD8 T cells^40,41^, have reduced capacity for the immunoregulatory killing of autologous activated T cells^40,50,51^ and are associated with greater expansion of virus-specific T cell responses^50,51^ and production of broadly neutralizing antibodies in HIV-1 infection^52^. Thus, by virtue of these unique immunological features, adaptive NK cells could augment humoral and T cell responses and/or provide a complementary layer of protection through antibodies that can mediate ADCC, especially in PLWH with suboptimal neutralizing antibody responses^5,6^. Adaptive/memory-like NK cells have been reported in patients with COVID-19 disease, suggesting that these NK cell subpopulations may arise in response to SARS-CoV-2 infection^14,48,53,54^. Interestingly, genetic deletion of NKG2C is a risk factor for severe SARS-CoV-2 infection, underscoring the potential protective role of adaptive NK cells in SARS-CoV-2 infection^55^. However, the impact of SARS-CoV-2 infection on NK cells in PLWH and their contribution to vaccine-mediated responses has not been previously investigated.

To bridge this knowledge gap, we performed a comprehensive assessment of NK cell phenotypic changes, evaluated their relationship to T cell and antibody measures and breadth and assessed their Fc-dependent responses to Spike in a well-characterized cohort of PLWH in the convalescent phase of SARS-CoV-2 infection and post-vaccination. Our findings show that adaptive NK cell subsets contribute to vaccine-mediated responses in PLWH, suggesting that this population can be promoted to potentiate vaccine efficiency.

## Results

### Clinical cohort

Thirty PLWH, the majority of whom were on antiretroviral therapy (ART) with an undetectable (< 50 copies/ml) HIV-1 viral load (94%), were recruited to this study following lab-confirmed SARS-CoV-2 diagnosis (RT-PCR + and/or antibody positive)^56^. Two individuals had detectable HIV viremia at the time of recruitment, 1549 copies/ml and 2.5 million copies/ml (CD4^+^ T-cell count: 92 and 130 cells/mm^3^, respectively). The cohort included participants with a median CD4^+^ T-cell count of 560 cells/mm^3^ (range 92–1110 cells/mm^3^) and a median CD4:CD8 ratio of 0.92 (range 0.12–2.54). All participants were recruited during the convalescent phase of SARS-CoV-2 infection with a median of 148 days post-symptom onset (DPSO) (range 46–232 days). The majority of these individuals had mild infection (score 1–2 on WHO criteria), and six donors experienced moderate disease requiring hospitalization (score 4–5 on WHO criteria). Twenty-eight age- and sex-matched PLWH, with no prior exposure (SARS-CoV-2 immunoglobulin G (IgG) seronegative for Spike (S1) and Nucleoprotein (N))^56^ at the time of sampling, were included as controls (SARS-CoV-2 naïve). The majority of SARS-CoV-2 naïve PLWH were on ART (89%) with a similar median CD4^+^ T-cell count of 573 cells/mm^3^ (range: 20–1029 cells/mm^3^) and a median CD4:CD8 ratio of 1.02 (range 0.3–2.26). Three SARS-CoV-2 naïve donors had a detectable viral load of 131, 25,704, and 1.8 × 10^6^ copies/ml (CD4^+^ T-cell count: 730, 480, and 130 cells/mm^3^, respectively). A subset of these participants with prior SARS-CoV-2 infection (n = 12) or SARS-CoV-2 naïve (n = 19) were followed up longitudinally after one, two, or three doses of SARS-CoV-2 immunization. All participants were HCMV-seropositive and predominately white males, with a median age of 51 years (range 31–72). Further details on patients’ characteristics and co-morbidities are included in Supplementary Table 1.

### Increased frequencies of adaptive NK cells following SARS-CoV-2 infection in PLWH

**Figure 1:**
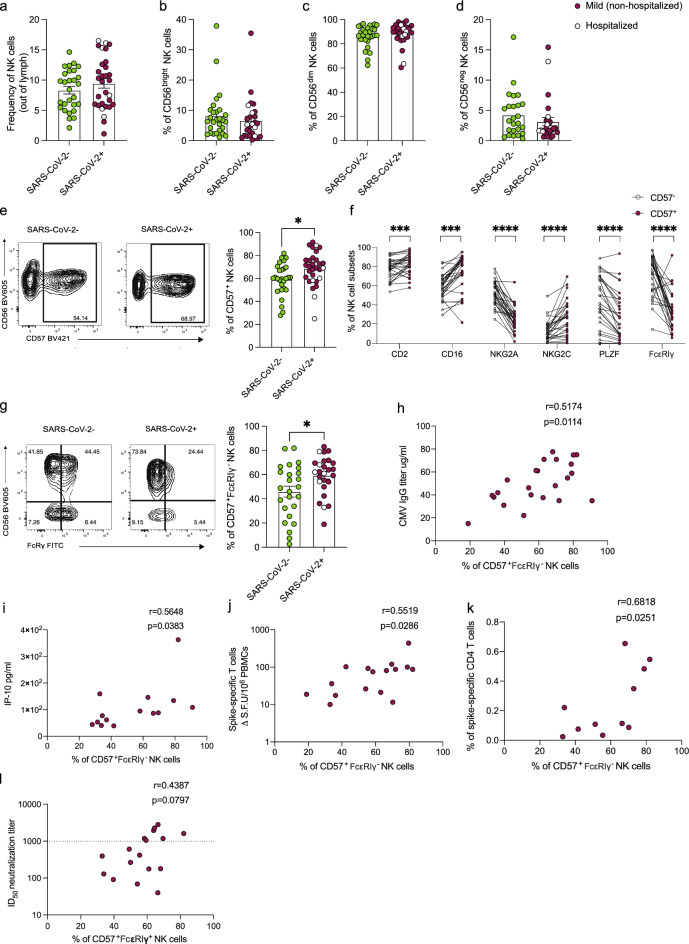
Phenotypic assessment of NK cells in convalescent SARS-CoV-2 infection in PLWH. Summary analysis of the frequency of (**a**) total NK cells out of lymphocytes, (**b**) CD56^bright^, (**c**) CD56^dim^, and (**d**) CD56^neg^ NK cells in SARS-CoV-2 negative (n = 28, green) and SARS-CoV-2-positive (n = 30, red) PLWH. Filled red dots: mild (non-hospitalized cases); Open circle: hospitalized cases. (**e**) Representative flow cytometric plots and summary analysis of the percentage of CD57^+^ NK cells in SARS-CoV-2-positive and -negative PLWH. (**f**) Paired analysis showing the expression of CD2, CD16, NKG2A, NKG2C, PLZF, and FcεRIγ markers within CD57^+^ (red) and CD57^−^ (white) NK cells in SARS-CoV-2-positive PLWH. (**g**) Representative plots and summary analysis of the percentage of FcεRIγ^+^CD57^+^ NK cells in the study groups. Correlation analysis between the frequency of FcεRIγ^+^CD57^+^ NK cells and (**h**) HCMV IgG titers and (**i**) plasma level of IP-10 in SARS-CoV-2-positive PLWH. Correlation analysis between the frequency of FcεRIγ^+^CD57^+^ NK cells and (**j**) magnitude of Spike-specific T cell responses measured by IFN-γ-ELISpot and (**k**) percentage of Spike-specific CD4^+^ T cells in SARS-CoV-2-positive PLWH. (**l**) Correlation between frequencies of FcεRIγ^+^CD57^+^ NK cells in SARS-CoV-2-positive PLWH and neutralization titers (ID_50_) against SARS-CoV-2 pseudovirus. Data are represented as geometric mean ± SEM [(**a**), (**b**), (**c**), (**d**), (**e**), (**g**), (**l**)]. Significance determined by two-tailed Mann–Whitney U test [(**a**), (**b**), (**c**), (**d**), (**e**), (**g**), (**l**)] or Wilcoxon-signed rank test [(**f**)]; *p < 0.05, **p < 0.01, ***p < 0.001, ****p < 0.0001. Non-parametric Spearman test (two-tailed) was used for correlation analysis (unadjusted *p* value displayed) [Adjusted *p* values after Benjamini-Hochberg (**h**, *p* = 0.0214), (**i**, *p* = 0.0478), (**j**, *p* = 0.0429), (**k**, *p* = 0.0418)]. See also Supplementary Fig. 1.

NK cell depletion and/or subset redistribution have been previously reported during acute SARS-CoV-2 infection in the general population, but these changes largely recover in the convalescent phase^11,14,57–59^. We, therefore, analyzed the percentage of total NK cells and the main NK cell subsets (CD56^bright^, CD56^dim^, and CD56^neg^CD16^+^ NK cells) in the study groups. We did not detect any changes in the total NK cell frequencies or frequencies of the main NK cell subsets (CD56^bright^, CD56^dim^, and CD56^neg^CD16^+^) in PLWH with or without prior SARS-CoV-2 infection (Fig. 1a–d and Supplementary Fig. 1a). Similarly, there were no significant differences in the percentage of activated (CD38^+^) or cytotoxic (GranzymeB^+^CD56^bright^) NK cells, which have been previously reported to expand during acute SARS-CoV-2 infection^14^ (Supplementary Fig. 1b and c). The degree of NK cell differentiation was evaluated by the expression levels of NKG2A and CD57 (denoting maturation). We noted variability in the levels of expression of CD57 in NK cells, with PLWH with prior SARS-CoV-2 infection showing a higher mean level of expression of CD57 and overall higher proportions of more differentiated CD57^+^ NK cells, compared to SARS-CoV-2 naïve PLWH (Fig. 1e). These differences were however modest, and should be interpreted with consideration of our cohort, primarily consisting of PLWH recovering from mild SARS-CoV-2 infection limiting any potential attributions to disease severity. No differences were observed in the percentage of NKG2A^+^ NK cells between the two groups (Supplementary Fig. 1d). Further phenotypic characterization of the CD57^+^ compared to their CD57^−^ NK cell counterparts, demonstrated enrichment of several adaptive NK cell markers within CD57+ cells, including lower levels of FcεRIγ and PLZF, and higher expression of NKG2C and CD2 (Fig. 1f). Specifically, an increased frequency of FcεRIγ^−^CD57^+^NK cells, and a trend towards higher frequencies of NKG2C^+^CD57^+^NK cells, were observed in SARS-CoV-2-positive compared to SARS-CoV-2 naïve PLWH (Fig. 1g, Supplementary Fig. 1e). These findings are consistent with FcεRIγ-deficient adaptive NK cells, which are often associated with HCMV infection and can expand upon exposure to antibody-coated targets and/or immune complexes^47–49^. In this respect, there was a trend towards lower CD16 expression and CD16 MFI in total NK cells in PLWH with prior SARS-CoV-2 infection compared to SARS-CoV-2 naïve PLWH (Supplementary Fig. 1f and g).

We next examined the relationship between FcεRIγ^−^CD57^+^NK cells, antibody levels, and circulating inflammatory markers in our cohort. The percentage of adaptive NK cells (FcεRIγ^−^CD57^+^NK cells) correlated positively with HCMV IgG titers (r = 0.5174 *p* = 0.0114) and weakly with the levels of CXCL10 (IP-10) (r = 0.5648 *p* = 0.0383) in PLWH with prior SARS-CoV-2 infection (Fig. 1h and i) but did not correlate with SARS-CoV-2 S1 or N IgG-specific binding titers (Supplementary Fig. 1h and i); the relationship between HCMV IgG titers and FcεRIγ-CD57 + NK cell frequencies was similar in SARS-CoV-2 naïve PLWH but did not reach statistical significance (Supplementary Fig. 1j). Overall, no significant differences were observed in the levels of HCMV IgG titers and CXCL10 between the two groups (Supplementary Fig. 1k and l).

Given the ability of NK cells to modulate T cells and neutralizing antibody responses, we evaluated the association between adaptive NK cell subsets and virus-specific responses^50,60–63^. We observed a weak positive correlation between the percentage of FcεRIγ^−^CD57^+^ NK cells and the magnitude of Spike-specific T cells, measured by IFN-γ ELISpot (r = 0.5519, *p* = 0.0286) and Spike-specific CD4 T cells, which we have previously shown to dominate SARS-CoV-2-specific T cell responses in convalescent PLWH (r = 0.6818, *p* = 0.0251) (Fig. 1j and k)^64^. A trend towards a positive correlation was observed between the frequencies of FcεRIγ^−^CD57^+^ NK cells and levels of SARS-CoV-2 neutralizing antibodies, with PLWH with higher frequencies of FcεRIγ^−^CD57^+^ NK cells tending to exhibit more potent neutralization levels (ID_50_ > 1000) (Fig. 1i). Taken together, while considering the limitations of a relatively modest cohort size, these data suggest that SARS-CoV-2 infection could contribute to increased NK cell maturation and adaptiveness in convalescent PLWH. These changes in the NK cell compartment could play a role in shaping humoral and cellular SARS-CoV-2 specific responses.

### Longitudinal assessment of NK cells in PLWH following SARS-CoV-2 vaccination

**Figure 2:**
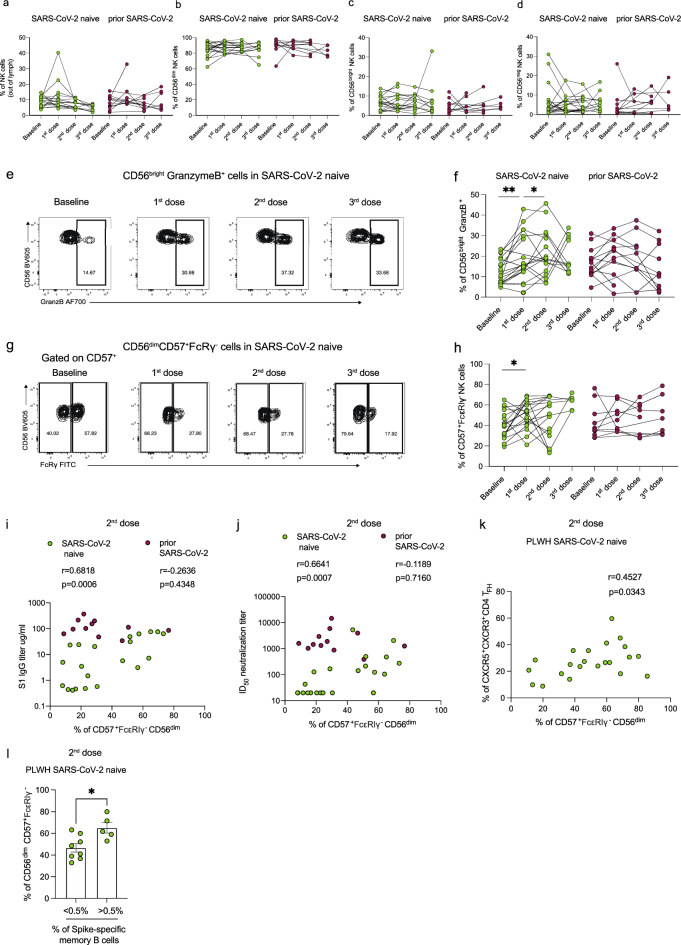
Longitudinal assessment of adaptive NK cells following SARS-CoV-2 vaccination in PLWH. Longitudinal analysis of the frequencies of (**a**) total NK cells, (**b**) CD56^bright^, (**c**) CD56^dim^, and (**d**) CD56^neg^ NK cells in PLWH with or without prior SARS-CoV-2 infection at baseline (pre-vaccine) and following first, second, and third dose of vaccine. (**e**) Representative flow cytometric plots and (**f**) summary analysis of the percentage of GranzB^+^CD56^bright^ NK cells. (**g**) Representative flow plots and (**h**) summary analysis of the percentage FcεRIγ^+^CD57^+^ CD56^dim^ NK cells. Correlation analysis between the frequency of FcεRIγ^+^CD57^+^ NK cells and (**i**) S1 IgG titers or (**j**) neutralizing titers (ID_50_) after two doses of vaccine in PLWH with or without prior SARS-CoV-2 infection. (**k**) Correlation analysis between percentage of FcεRIγ^+^CD57^+^ NK cells and CXCR3^+^CXCR5^+^T_FH_ cells after two doses of vaccine in SARS-CoV-2 naïve PLWH. (**l**) Comparison of the percentage of FcεRIγ^+^CD57^+^ NK cells in SARS-CoV-2 naïve PLWH according to their levels of Spike-specific memory B cells. The threshold was set to 0.5%. Significance determined by two-tailed Wilcoxon-signed rank test [(**a**), (**b**), (**c**), (**d**), (**f**), (**h**)], or two-tailed Mann–Whitney U test [(**i**)]; *p < 0.05, **p < 0.01. Non-parametric Spearman test (two-tailed) was used for correlation analysis (unadjusted *p* value displayed) [Adjusted *p* values after Benjamini-Hochberg (**i**, SARS-CoV-2 naïve *p* = 0.0012), (**j**, SARS-CoV-2 naïve *p* = 0.0014), (**k**, SARS-CoV-2 naïve *p* = 0.0686)] Data are represented as geometric mean ± SEM [(**i**)]. See also Supplementary Fig. 2.

The increased proportion of terminally differentiated adaptive NK cells following natural SARS-CoV-2 infection prompted us to evaluate whether similar changes to the NK cell pool are observed following SARS-CoV-2 vaccination. We, therefore, performed a longitudinal analysis of the NK cell frequencies and phenotype in a subset of PLWH with or without prior SARS-CoV-2 infection following vaccination. There was no significant difference in the frequency of total NK cells, or the percentage of CD56^bright^, CD56^dim^, and CD56^neg^CD16^+^ NK cell subsets, following SARS-CoV-2 vaccination (post-first, -second, and -third dose) in both study groups compared to baseline (Fig. 2a–d). Further phenotypic evaluation revealed increased frequencies of GranzymeB^+^CD56^bright^ NK cells in SARS-CoV-2 naïve PLWH after one or two vaccine doses, reaching a level comparable to that observed in individuals with prior infection (Fig. 2e and f). Additionally, we observed a higher percentage of FcεRIγ^−^CD57^+^ CD56^dim^ NK cells in SARS-CoV-2 naïve PLWH following the first dose of vaccine, consistent with changes observed in the convalescent phase of SARS-CoV-2 infection in PLWH (Fig. 2g and h). There was no significant effect of vaccination on the frequencies of NKG2C^+^CD57^+^CD56^dim^ NK cells (Supplementary Fig. 2a).

We recently reported attenuated humoral responses following vaccination in PLWH^5^. Given the potential role of NK cell subsets to modulate antibody responses^13,52,65,66^, we related our phenotypic findings to the magnitude of antibody binding titers. The proportion of FcεRIγ^−^CD57^+^ NK cells correlated positively with vaccine-induced S1 IgG titers after one dose of vaccine in PLWH with prior SARS-CoV-2 infection (r = 5659, *p* = 0.0473) but not in SARS-CoV-2 naïve individuals (due to the low antibody titers in SARS-CoV-2 naïve group) (Supplementary Fig. 2b); however, after two doses of vaccine, a significant association was observed between FcεRIγ^−^CD57^+^ NK cells and S1 IgG titers in SARS-CoV-2 naïve PLWH (r = 0.6818, *p* = 0.0006) (Fig. 2i). Similarly, there was a positive correlation between FcεRIγ^−^CD57^+^ NK cells and neutralizing antibody titers after two vaccine doses in PLWH SARS-CoV-2 naïve (r = 0.6641, *p* = 0.0007) (Fig. 2j). No correlation was observed between FcεRIγ^−^CD57^+^ NK cells, S1 IgG, and neutralizing antibody titers after three doses of vaccine in both study groups (Supplementary Fig. 2c and e).

For cellular responses, Spike-specific T cell responses correlated positively with the proportion of FcεRIγ^−^CD57^+^ NK cells after one dose of vaccine in PLWH with prior infection but not in SARS-CoV-2 naïve PLWH; however, there was a trend towards a positive association after two doses of vaccine in SARS-CoV-2 naïve PLWH (Supplementary Fig. 2f. and g). No significant associations were detected after three doses of vaccine in both study groups (Supplementary Fig. 2h). Consistent with NK cell modulation of T_FH_ cell responses, we observed a trend towards a positive correlation between the percentage of FcεRIγ^−^CD57^+^ NK cells and T_FH_ cell frequencies (r = 0.4527, *p* = 0.0343 “adjusted *p* = 0.0686”) (Fig. 2k), suggesting that these cells may influence the development of neutralizing antibodies through less negative regulation of T_FH_ cell responses supplying help to B cells^52,67–69^. Supporting this notion, a higher proportion of FcεRIγ^−^CD57^+^ NK cells was observed in PLWH with higher frequencies of virus-specific memory B cells (> 0.5%) (Fig. 2l). Taken together, these findings suggest that vaccination leads to NK cell activation and changes in the NK cell pool that could influence the development/magnitude of humoral and cellular responses in PLWH.

### Antibodies induced by SARS-CoV-2 vaccination can trigger antibody-mediated NK cell responses in PLWH

**Figure 3:**
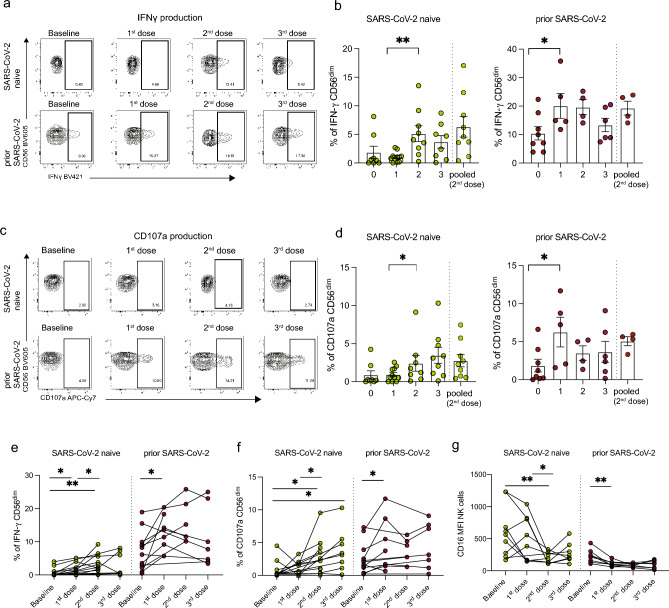
Antibody-dependent NK cell responses following SARS-CoV-2 vaccination in PLWH. (**a**) Representative flow cytometric plots and (**b**) cross-sectional analysis of the percentage of IFN-γ^+^ CD56^dim^ NK cells from PLWH, with or without prior SARS-CoV-2 infection, at baseline (pre-vaccine) and following each dose of vaccine. (SARS-CoV-2 naïve: n = 14 baseline, n = 9 first dose, n = 12 s dose, n = 8 third dose; prior SARS-CoV-2 n = 12 baseline, n = 7 first dose, n = 5 s dose, n = 7 third dose). (**c**) Representative flow cytometric plots and (**d**) cross-sectional analysis of frequencies of CD107a^+^ CD56^dim^ NK cells. Longitudinal analysis of (**e**) IFN-γ^+^ CD56^dim^ NK cells, (**f**) CD107a^+^CD56^dim^ NK cells, and (**g**) CD16 MFI in the two study groups. Significance determined by two-tailed Mann–Whitney U test [(**b**), (**d**)], or two-tailed Wilcoxon-signed rank test [(**e**), (**f**), (**g**)]; *p < 0.05, **p < 0.01. Data are represented as geometric mean ± SEM [(**b**), (**d**)]. See also Supplementary Fig. 3.

Next, we sought to investigate the functional consequences of the observed phenotypic differences in NK cell subsets in PLWH with or without prior SARS-CoV-2 infection following vaccination. Given the importance of ADCC in the control of SARS-CoV-2^22,27^, we established an assay to analyze the Fc receptor-dependent activation of NK cells in response to anti-SARS-CoV-2 antibody stimulation (Supplementary Fig. 3a). PBMCs from PLWH were cultured with plate-bound Spike in the presence of autologous pre- and post-vaccination serum samples. NK cell activation was measured in response to autologous serum collected at baseline (pre-vaccine), post-first, -second, and -third dose plus immobilized Spike in each study participant (SARS-CoV-2 naïve n = 14, prior SARS-CoV-2 n = 12). Pre-pandemic (serum samples collected before 2018) or pooled serum (following two doses of vaccine) were used as controls. As expected, PLWH with prior SARS-CoV-2 infection had greater levels of antibody-dependent NK cell activation, measured by IFN-γ and CD107 expression, at baseline (pre-vaccine) compared to SARS-CoV-2 naïve PLWH (Fig. 3a–d). Significantly higher frequencies of NK cells expressing IFN-γ and CD107a were observed post-second dose in individuals with no prior SARS-CoV-2 infection and following the first vaccine dose in PLWH with prior exposure (Fig. 3a–d). Notably, responses to second-dose autologous serum were comparable in magnitude to the NK cell responses detected after stimulation with pooled serum representing a ‘constant’ antibody concentration (Fig. 3a–d).

We examined the evolution of these responses following SARS-CoV-2 vaccination in longitudinal samples (SARS-CoV-2 naïve n = 14, prior SARS-CoV-2 n = 12). The frequencies of NK cells producing IFN-γ in response to post-first dose serum were higher than in response to pre-vaccination (baseline); these responses were further increased post-second dose autologous serum reaching a plateau post-third dose in SARS-CoV-2 naïve PLWH (Fig. 3e). Unlike IFN-γ production, NK cell degranulation, measured by CD107a, was induced only after two doses of vaccine in SARS-CoV-2 naïve individuals and was further boosted by a third dose (Fig. 3f). In contrast, PLWH with prior SARS-CoV-2 infection demonstrated higher IFN-γ and CD107a responses at baseline, with a boosting effect observed only after the first dose (Fig. 3e and f). Similar effects were observed for TNF-α production (Supplementary Fig. 3b and c). The levels of IFN-γ produced by NK cells in SARS-CoV-2 naïve PLWH remained lower despite three vaccine doses compared to SARS-CoV-2 exposed PLWH (Fig. 3e). In keeping with the observed changes in NK cell function following vaccination, CD16 expression, measured by the median MFI, was lower in response to post-second or -third dose serum compared to baseline in PLWH with no prior SARS-CoV-2 infection and following the first dose in PLWH with prior exposure (Fig. 3g).

Next, we assessed whether samples collected from PLWH prior to the COVID-19 pandemic contained cross-reactive antibodies and if these pre-existing antibodies can induce NK cell-mediated ADCC activity against SARS-CoV-2^22,70^. Interestingly, NK cell responses were detected following incubation with pre-pandemic serum samples from two SARS-CoV-2 naïve PLWH (Supplementary Fig. 3d), suggesting that the presence of SARS-CoV-2 cross-reactive antibodies can mediate functional NK cell responses in PLWH individuals. Notably, the two donors with pre-existing NK cell responses to the pre-pandemic serum showed a higher response to autologous serum following two doses of SARS-CoV-2 vaccination compared to those without pre-existing responses (Supplementary Fig. 3e). These responses were enriched in adaptive (CD57^+^FcεRIγ^−^) NK cell subsets (Supplementary Fig. 3e and f). Together, these data show that SARS-CoV-2 infection and/or vaccination induces robust antibody-dependent NK cell responses to immobilized Spike protein in PLWH.

### Antibody-dependent NK cell responses are enriched in adaptive NK cell subsets

**Figure 4:**
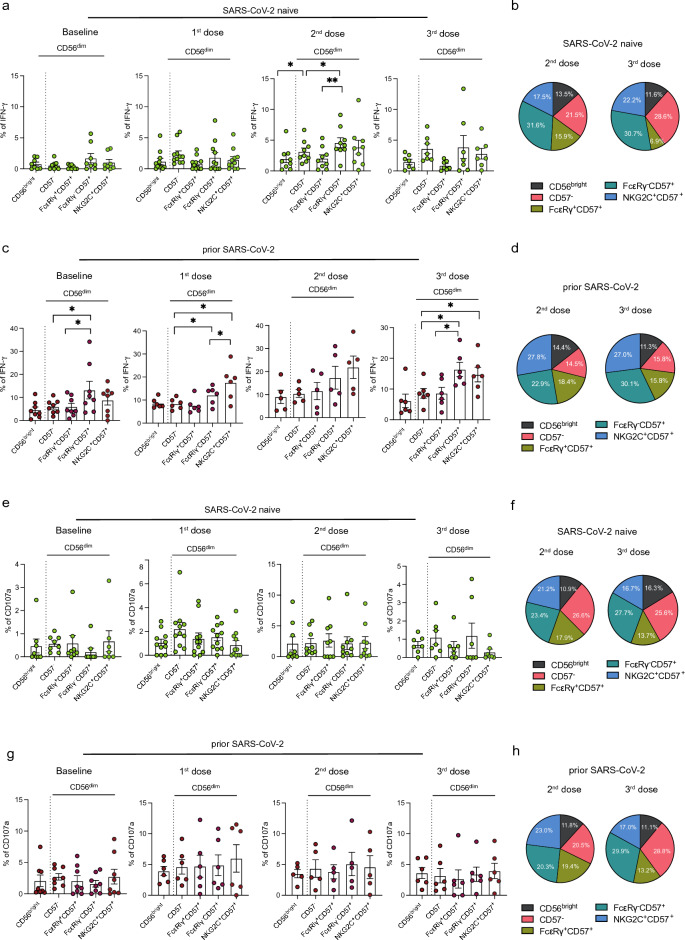
Antibody-dependent responses of different NK cell subsets in PLWH. (**a**) Summary analysis and (**b**) pie charts showing the percentage of IFN-γ^+^ within CD56^bright^, CD56^dim^CD57^−^, CD56^dim^CD57^+^FcεRIγ^+^, CD56^dim^CD57^+^FcεRIγ^−^, and CD56^dim^CD57^+^NKG2C^+^ cells in SARS-CoV-2 naïve PLWH at baseline and after one, two or three doses of vaccine (as indicated above each figure). NK cell subsets in the pie chart are color-coded in the key. (**a**) Summary analysis and (**b**) pie charts showing the percentage of IFN-γ^+^ within different NK cell subsets in PLWH with prior SARS-CoV-2 infection. (**e**) Summary analysis and (**f**) pie charts showing the percentage of CD107a^+^ within different NK cell subsets in SARS-CoV-2 naïve PLWH at baseline and after each vaccine dose. (**g**) Summary analysis and (**h**) pie charts representing the frequencies of CD107a^+^ within different NK cell subsets in PLWH with prior SARS-CoV-2 infection. Significance determined by two-tailed Wilcoxon-signed rank test [(**a**), (**c**), (**e**), (g)]; * p < 0.05, ** p < 0.01. Data are represented as geometric mean ± SEM [(**a**), (**c**), (**e**), (**g**)]. See also Supplementary Fig. 4.

To evaluate the influence of NK cell phenotype variation on the magnitude of antibody-dependent NK cell function, we analyzed the functional capacity of specific NK cell subsets. IFN-γ responses were assessed in CD56^bright^, CD57^−^, CD57^+^NKG2C^+^ CD56^dim^, and CD57^+^FcεRIγ^+^ CD56^dim^ NK cell subsets in PLWH with or without prior SARS-CoV-2 infection (Fig. 4a and b, Supplementary Fig. 4a). In SARS-CoV-2 naïve PLWH, IFN-γ expression after two vaccine doses was more strongly mediated by CD57^+^FcεRIγ^−^ compared to CD57^−^ subsets with almost half of the total IFN-γ production attributed to adaptive NK cells (CD57^+^FcεRIγ^−^ and CD57^+^NKG2C^+^ CD56^dim^ NK cells) (Fig. 4a and b). Given the low-level antibody responses after the first vaccine dose in SARS-CoV-2 naïve PLWH, no significant differences in IFN-γ expression between different NK cell subsets were detected (Fig. 4a). Similarly, IFN-γ production was significantly enriched within adaptive/differentiated CD57^+^FcεRIγ^−^ and CD57^+^NKG2C^+^ subsets in PLWH with prior infection across all timepoints analyzed (Fig. 4c and d). In contrast to IFN-γ expression, no significant differences were observed in the levels of CD107a expression between different NK cell subsets (CD56^bright^ vs. more differentiated subsets) in PLWH with or without prior SARS-CoV-2 infection (Fig. 4e–h, Supplementary Fig. 4b). Together, these data demonstrate a stronger antibody-dependent response in terms of cytokine production mediated by more differentiated adaptive NK cells.

**Figure 5:**
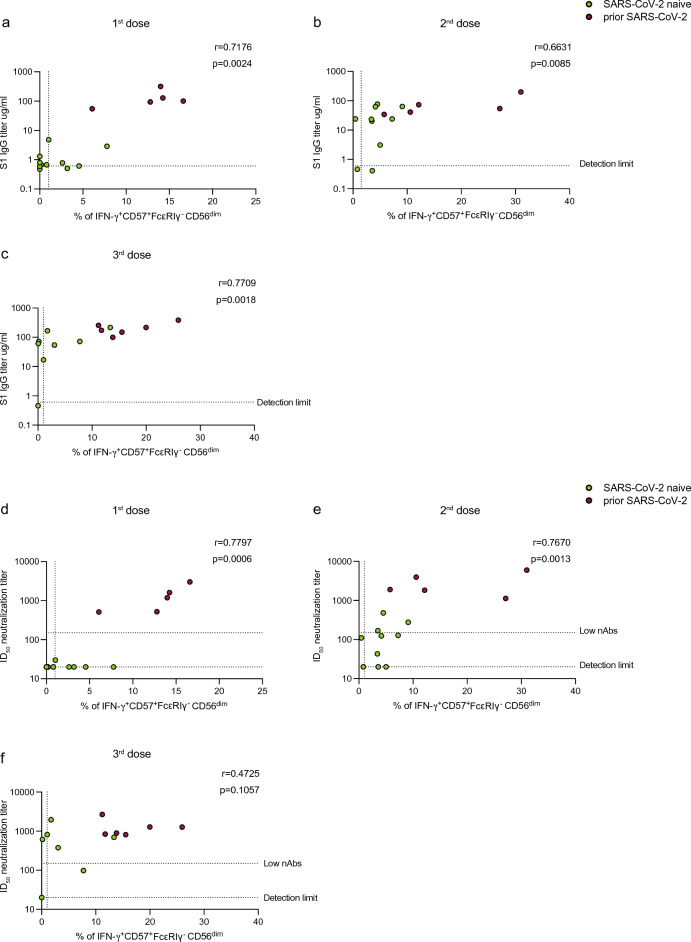
Correlation analysis between antibody-dependent NK cell responses and anti-SARS-CoV-2 antibody concentration in PLWH. Correlation of IFN-γ^+^ CD57^+^FcεRIγ^−^ CD56^dim^ NK cell responses with S1 IgG-specific titers after (**a**) first, (**b**) second, and (**c**) third dose in PLWH, with or without prior SARS-CoV-2 infection (limit of detection 0.6 µg/ml). Correlation between the frequencies of IFN-γ^+^ CD57^+^FcεRIγ^−^ CD56^dim^ NK cells and nAb titers after (**d**) first, (**e**) second, and (**f**) third dose in PLWH, with or without prior SARS-CoV-2 infection (limit of detection ID_50_ = 20, low level of nAb ID_50_ = 150). The non-parametric Spearman test was used for correlation analysis (two-tailed) (unadjusted *p* value displayed) [Adjusted *p* values after Benjamini-Hochberg (**a**, *p* = 0.006), (**b**, *p* = 0.0182), (**c**, *p* = 0.003), (**d**, *p* = 0.0035), (**e**, *p* = 0.006), (**f**, *p* = 0.0048)].

We evaluated the relationship between SARS-CoV-2 antibody titers and adaptive NK cell functional responses by comparing antibody and neutralization levels with the frequency of IFN-γ^+^CD57^+^FcεRIγ^+^CD56^dim^ cells following SARS-CoV-2 vaccination. Overall, antibody-dependent NK cell responses (IFN-γ^+^CD57^+^FcεRIγ^+^CD56^dim^) following the first, second, and third vaccine doses correlated positively with S1 IgG binding titers (first dose r = 0.7176, *p* = 0.0024; second dose r = 0.6631, *p* = 0.0085; third dose r = 0.7709, *p* = 0.0018) (Fig. 5a–c) and neutralizing antibody titers (first dose r = 0.7797, *p* = 0.0006; second dose r = 0.7670, *p* = 0.0013; third dose r = 0.4725, *p* = 0.1057) in PLWH with or without prior SARS-CoV-2 infection (Fig. 5d–f). Despite these positive associations, some PLWH, who lacked neutralizing antibody response and/or had low levels of neutralization (ID_50_ < 150), had measurable antibody-dependent NK cell responses (Fig. 5d–f).

## Discussion

NK cell subsets are emerging as attractive vaccine targets owing to their ability to regulate adaptive responses, develop recall responses to antigen re-stimulation and effectively synergize with vaccine-induced antibodies to moderate infection^11,13,71,72^. PLWH represent a unique group of patients to study the contribution of NK cell subsets to SARS-CoV-2 vaccination. NK cell subsets are strongly remodeled during HIV-1 infection, bearing adaptive traits with retained functional capacity^36,45^ that can modify the evolution of subsequent immune responses^13,72,73^. Our data support the hypothesis that the adaptive NK cell compartment in PLWH, partly driven by HCMV co-infection, can be re-shaped by SARS-CoV-2 infection and/or vaccination, leading to a more differentiated adaptive phenotype. These phenotypic alterations could subsequently influence NK cell antibody-dependent responses and the generation of SARS-CoV-2 specific cellular and humoral immunity. Further research is required to evaluate the extent to which these adaptive NK cell expansions represent the effect of CMV reactivation, the direct effect of SARS-CoV-2 infection/vaccination and/or the potential cumulative influence of these factors within the pre-existing and variable pool of adaptive NK cells. Nonetheless, these observations provide valuable insights for the manipulation of specific NK cell subsets through novel vaccination strategies in favor of populations with stronger ADCC capacity. While the relevance of these findings extends to PLWH, their broader implications warrant consideration and should be validated within larger cohorts that include PLWH and HIV-negative individuals.

Our findings showed that PLWH with prior exposure to SARS-CoV-2 had a larger proportion of mature/differentiated NK cells that lacked expression of FcεRIγ. These CD57^+^FcεRIγ^−^ subsets were detected in at least 148 DPSO following predominantly mild infection and were induced post-vaccination in SARS-CoV-2 naïve PLWH. In the context of convalescent infection, the presence of CD57^+^FcεRIγ^−^ populations was associated with HCMV IgG and CXCL10 (IP-10) levels. We and others have previously described that these subsets in HIV-1 and other viral infections are partly driven by HCMV co-infection/reactivation and the level of pro-inflammatory cytokines^39,52,74,75^. Although it is possible that some of these changes could reflect the impact of HIV-1 on NK cell repertoire^76–78^, these data are also consistent with the expansion of adaptive NK cells during COVID-19 infection in HCMV-seropositive HIV-negative individuals^14,48,53,54^. Increased maturation and NK cell ‘adaptiveness’ could reflect the distinct microbial stimuli/exposure and/or epigenetic remodeling following an acute infection or vaccination ^40–42,79–81^. Although the expansion of adaptive FcεRIγ^−^ NK cells is associated with HCMV co-infection and higher levels of expression of NKG2C, including in healthy individuals’ peripheral blood and tissues^82,83^, these populations are not exclusively confined to NKG2C^+^ NK cells. Adaptive FcεRIγ^−^ NK cells can be expanded ex vivo following CD16 stimulation through interactions with CD3z homodimers^40,41,47^. In line with these observations, we observed a trend towards lower CD16 expression in PLWH who recovered from COVID-19 disease. These subpopulations have been reported during COVID-19 disease, corresponding to higher TGFβ and IFNα levels and potential alterations in signaling pathways^48^. Interestingly, while previous reports have shown that the expansion of adaptive NK cells in patients with acute SARS-CoV-2 infection was confined to HCMV seropositive donors, this was independent of HCMV reactivation^14^. It is, therefore, likely that the accumulation of adaptive NK cells observed in our SARS-CoV-2 convalescent cohort is triggered by multiple factors. These NK cell subsets could result from pre-existing populations following slow homeostatic proliferation mediated by potential HCMV reactivation during heterologous infection or cross-reactive antigens in addition to signals received by CD16 and/or cytokine priming^40,41,48,49^.

Of note, our results reflect changes in the NK cell compartment during the convalescent phase and do not exclude any initial changes during acute SARS-CoV-2 infection. Additionally, NK cell-specific memory responses have been reported in the context of other viral infections, including HIV-1 and influenza, and following vaccination with Hepatitis B (HBV) antigens^79–81,83^, and NKG2C^+^CD57^+^NK cells from convalescent donors have been shown to produce IFN-γ in response to soluble SARS-CoV-2 peptides^53^. Future longitudinal studies are required to dissect the mechanisms underlying the dynamic adaptations of NK cells in response to SARS-CoV-2 infection/vaccination in PLWH and examine for any NK cell SARS-CoV-2 specific recognition. A better understanding of the memory-like functionality of NK cells could further guide new vaccine strategies (NCT02416453)^84^.

The adaptive NK cell subsets described in this study are strongly antibody reactive^41,47,49,85^. Durable memory-like NK cell responses have been described following a heterologous prime-boost viral vector-based Ebola virus vaccine, resulting in persistent antibody-dependent NK cell activation up to 180 days after the booster dose^86^. Interestingly, the presence of adaptive NK cells in healthy HCMV seropositive individuals significantly modified responses to Ebola glycoprotein^87^. Consistent with these findings, our data show that a durable adaptive NK cell response was detected up to 148 days post-infection, or 218 days post-second dose of vaccination, suggesting that these cells could potentially contribute to long-lasting immunity. While virus neutralization represents an important correlate of protective antiviral immunity, other non-neutralizing Fc effector functions mediated by antibodies, including NK cell-mediated ADCC, could, therefore, contribute to viral control, especially against emerging variants^27^. In keeping with this, we observed a superior capacity for antibody-dependent responses against immobilized Spike in the presence of autologous pre- and post-vaccination serum, mediated by adaptive subsets relative to less differentiated NK cells. Unsurprisingly, individuals with prior exposure had higher baseline IFN-γ production, which was further boosted via vaccination and exceeded responses in SARS-CoV-2 naïve PLWH even after three vaccine doses. However, CD107a responses were similarly induced in all NK cell subsets. This is in line with hypomethylated regulatory regions in IFN-γ and the interaction of FcεRγ with CD3z homodimers in adaptive NK cell subsets, providing the mechanisms for stronger induction of cytokine production upon Fc-dependent stimulation^40,47,88^. Notably, the magnitude of NK cell activation in response to autologous or pooled serum was comparable in both groups suggesting that heterogeneity in responses does not solely depend on antibody concentration and could reflect individual variation in the composition of the adaptive NK cell pool. Interestingly, in a small proportion of SARS-CoV-2 naïve PLWH with low or absent neutralizing antibodies after vaccination, we detected Fc-dependent NK cell responses, suggestive of the potential functional contribution of NK cells to vaccine-mediated protection that merits further investigation in larger cohorts.

In addition to their potential to mediate ADCC activity, adaptive NK cells have a reduced ability for immunoregulation and suppression of activated T cells^40^ and can modulate the induction of broadly neutralizing antibodies in HIV-1 infection through the reduced killing of T_FH_ T cells^52^. The observed relationships between adaptive NK cells and T cells and magnitude of humoral responses in this study would be consistent with these reports. However, to reinforce these findings and explore potential mechanisms additional investigation is essential in more extensive patient groups. These adaptive NK cell populations may have the added benefit of supporting the induction of B and T cell responses through direct interactions and/or secretion of cytokines^50^, which can contribute to virus-specific T cell expansions and recruitment to the site of infection^50,62,89^. NK cell subsets lacking FcεRIγ have been reported to augment CD8 T cell responses during lymphocytic choriomeningitis virus (LCMV) infection and contribute to the rapid control LCMV^51^. Mechanistically, FcεRγ is required to stabilize natural cytotoxicity receptors (NCRs) that are involved in killing CD4 and CD8 T cells and limiting antiviral T cell responses^51,63,90^. The absence of FcεRγ in adaptive NK cells charactreized by a significant reduction in NCR expression, could therefore result in augmented T cell responses^49,51^. Additionally, the bias towards production of activating cytokines/chemokines by adaptive NK cells, could recruit other immune cells to the site of infection, promoting virus-specific responses^50^. Another possibility is that the more efficient viral control by adaptive NK cells could balance the intensity of responses by other innate immune cells, encouraging optimal adaptive immune responses^91^.

These results are in keeping with recent observations showing that an increased frequency of NKG2C^+^ NK cells correlates with high antibody titers following BNT162b2 mRNA vaccination^54^. Although the full impact of HCMV co-infection on vaccine responses can be broad (reviewed in^92^), our data suggest that the increased NK cell differentiation and presence of adaptive features in PLWH could be advantageous, favoring antibody-mediated activation and induction/preservation of T cell and humoral responses. Further research and mechanistic experiments are needed to validate these observations in healthy and/or older individuals according to HCMV serostatus to refine our understanding of NK cell contribution to vaccine efficacy.

SARS-CoV-2 vaccination also induced an expansion of GranzymeB^+^CD56^bright^cells in PLWH with no prior SARS-CoV-2 infection. These ‘armed’ peripheral blood CD56^bright^ NK cells have been described in hospitalized patients with ongoing SARS-CoV-2 viremia and severe disease and shown to correlate with IL-6 levels^14^. Activation and proliferation of cytotoxic CD56^bright^ NK cells have been previously reported as a feature of live-attenuated vaccines^86,87,93–95^. These findings suggest that common/shared soluble factors/signaling pathways during acute infection or post-vaccination could endow CD56^bright^ NK cells with cytotoxic potential and the ability to degranulate following antibody-dependent activation.

Our study has several limitations. It was not possible to study early NK cell responses during acute infection or during the first few days following vaccination. Due to the relatively small numbers in the study, it has not been possible to study the effect of different vaccine platforms and the ideal prime–boost strategy to elicit optimal NK cell activation. Additionally, our analysis provides insights into NK cell responses up to three vaccine doses; future studies are required to assess the durability of these responses and their contribution/complementary role in the longer-term immunity in PLWH, including protection against breakthrough infections and severe disease. Our study focused on antibody-dependent activation against Spike, the main vaccine target. Nucleoprotein-specific IgG antibodies, as well as those against other proteins (ORF3a and Membrane), have been shown to activate NK cells in healthy subjects^22,24^, and their potential role in PLWH requires further investigation. Along these lines, it would be of interest to determine in greater detail NK cell cross-reactive activity against SARS-CoV-2 by other endemic coronavirus antibodies or glycan-reactive anti-HIV antibodies that have been recently reported to bind to Spike protein^95^. Our assay measuring antibody-dependent NK cell activation involved autologous NK cells, as the study focused on post-vaccination NK cell responses, rather than solely evaluating antibody quality. To address potential individual variations in NK cell activity that could impact overall antibody-dependent NK cell function, both autologous and pooled post-vaccination serum were used. This plate-bound assay has limitations due to its lower sensitivity and inability to fully represent cell–cell interactions. However, a recent study assessing NK cell responses in SARS-CoV-2 vaccinated individuals using both plate-bound and cell-based assays showed a strong correlation between responses measured by both methods^22^. This suggests that our experimental approach did not significantly influence the results presented in this study.

Collectively, this study demonstrated a robust and durable antibody-dependent NK cell response detectable for at least 148 days after natural infection. These responses were elicited by SARS-CoV-2 vaccination in PLWH with no prior exposure and further boosted in people with hybrid immunity. Antibody-dependent responses were mediated by mature adaptive NK cell subsets, highlighting the potential to selectively promote these features through vaccination to enhance protection.

## Methods

### Ethics statement

The protocols for the human study were approved by the local Research Ethics Committee (REC)—Berkshire (REC 16/SC/0265). The study complied with all relevant ethical regulations for work with human participants and conformed to the Helsinki declaration principles and Good Clinical Practice (GCP) guidelines, and all subjects enrolled in the study provided written informed consent.

### Study cohort

PLWH on ART were recruited post-SARS-CoV-2 infection and/or vaccination. PLWH with prior COVID-19 disease (n = 30) were sampled during the convalescent phase with a median number of days post-symptom onset (DPSO) of 148 days. Confirmed SARS-CoV-2 infection by SARS-CoV-2 PCR and/or Roche antibody tests was declared by the participants, who were asked to provide details on the timing and nature of their symptoms. In addition to the self-reporting symptoms, all participants were screened for antibodies against the external Spike and internal nucleoprotein antigens using ELISA to confirm prior infection^56^. European Centre for Disease Prevention (ECDC) criteria were used for the case definition of COVID-19 disease. The severity of COVID-19 disease was defined according to the WHO criteria. Twenty-eight demographically age- and sex-matched PLWH who didn’t report prior infection and were found to be SARS-CoV-2 IgG seronegative at the time of sampling were included for comparison.

A total of 31 PLWH (n = 12 with prior infection; n = 19 without prior infection) were recruited after the first dose (median of 20 days, range 6–85), second dose (median 32 days, range 5–218), and third dose (median 64 days, range 5–199) of COVID-19 vaccine. Participants received a mix of available SARS-CoV-2 vaccination (Pfizer-BioNTech BNT162b2; Moderna mRNA-1273 or AstraZeneca AZD1222) according to the Joint Committee on Vaccination and Immunization, UK, guidelines. At each visit, participants were asked to report any history of SARS-CoV-2 infection. Four SARS-CoV-2 naïve PLWH reported a breakthrough infection, and thus, any subsequent time points were included in the ‘prior SARS-CoV-2 infection’ group for analysis. Longitudinal samples were not available for all participants (Supplementary Table 1).

All participants were screened for CMV seropositivity by CMV IgG (enzyme-linked immunosorbent assay “ELISA”) or CMV-specific T cells (IFN-γ ELISpot assay) before inclusion. Participants were recruited at the Mortimer Market Centre for Sexual Health and HIV-1 Research and the Ian Charleson Day Centre at the Royal Free Hospital (London, UK) following written informed consent as part of a study approved by the local ethics board committee. Additional information about the demographic and clinical characteristics, vaccine type and sampling intervals can be found in Supplementary Table 1. Further details on the exact number of subjects utilized for each assay are indicated in the figure legends and the “Results” Section. To minimize inter-experimental variability and batch effects, matched cryopreserved samples were run in batches with inter-assay quality controls.

### Peripheral blood mononuclear cells (PBMC) and plasma sample preparation

Venous blood samples were collected in heparin tubes, and PBMCs were isolated using Ficoll gradient centrifugation, as previously described in^64^. Whole blood was transferred to conical tubes and centrifuged at 2000 × g for 5 min. Plasma samples were then collected, and the remaining blood was diluted with RPMI (Corning, Manassas, VA, USA). Diluted blood was then layered over an appropriate volume of Histopaque (Histopaque-1077 Cell Separation Medium, Sigma Aldrich, St. Louis, MO, USA) and centrifuged for 20 min at 500 × g at room temperature without brake. The PBMC layer was carefully removed, transferred to a fresh tube, and washed with RPMI. Cells were stained and counted using Automated Cell Counter (BioRad, Hercules, California, USA). Isolated PBMCs were then cryopreserved in a freezing medium containing 10% dimethyl sulfoxide (DMSO) (MP Biomedicals, LLC, Irvine, CA, USA) and 90% heat-inactivated fetal bovine serum (FBS) and stored in liquid nitrogen for further use.

### Semiquantitative S1 and N ELISA

Anti-Spike IgG titers were measured using an ELISA assay, as previously described^64^. A 96-half-well Maxisorp plate (Nalgene, NUNC International, Hereford, UK) was coated with 25 μl of S1 or N purified protein (kindly gifted by Peter Cherepanov, Francis Crick Institute) at 3 µg/ml in PBS overnight at 4 °C. Three columns were coated with 25 μl goat anti-human F(ab)′2 (1:1000) to generate an internal standard curve. Plates were then washed with PBS-T (0.05% Tween in PBS) and blocked for 1 h with assay buffer (5% milk powder PBS-T) at room temperature. Serum was then added to the antigen-coated wells at 1:50–1:1000 dilution (25 μl in assay buffer) and incubated for 2 h. Serial dilutions of known concentrations of IgG were added to the F(ab)′2 IgG-coated wells in triplicate. Following 2h incubation, wells were washed with PBS-T, and 25 μl alkaline phosphatase-conjugated goat anti-human IgG (Jackson ImmunoResearch) was added at a 1:1000 dilution in assay buffer, and incubated for 1 h at room temperature. Plates were then washed, and 25 μl of alkaline phosphatase substrate (Sigma Aldrich) was added. ODs were measured using a MultiskanFC (ThermoFisher) plate reader at 405 nm, and S1 & N-specific IgG titers interpolated from the IgG standard curve using 4PL regression curve-fitting on GraphPad Prism 9.

### Pseudovirus production and neutralization assays

To produce HIV-1 particles pseudotyped with SARS-CoV-2 Spike, 3 × 10^6^ HEK-293T cells were seeded in 10ml of complete DMEM Dulbecco’s Modified Eagle’s Medium (Gibco) supplemented with 10% FBS and 50μg/ml penicillin–streptomycin. Cells were then transfected with 9.1 µg of HIV-1 p8.91 packaging plasmid^96^, 9.1 µg of HIV-1 luciferase reporter vector plasmid^97^, 1.4 µg of wild type (Wuhan-hu1 strain) SARS-CoV-2 Spike plasmid and 60 µg of PEI-Max (Polysciences). After 48h of transfection, supernatants were harvested, filtered (0.45 µm filter), and either used directly in the assay or stored at − 80 °C. Neutralization assays were performed by incubating serial dilutions of patient serum with pseudovirus in a 96-well plate for 1 h. Following 1 h incubation, HeLa ACE-2 cells (kindly gifted by James E. Voss, Scripps Institute) were added (10,000 cells per 100 μl/well). After 48/72 h, supernatants were removed, and the cells were lysed; Brightglo luciferase substrate (Promega) was added to the plates, and RLU was read on a Glomax luminometer (Promega) as a proxy for infection. Measurements were performed in duplicate, and 50% inhibitory dilution (ID_50_) values were then calculated using GraphPad Prism 9.

### NK cell phenotypic flow cytometric analysis

The list of fluorochrome-conjugated antibodies used in this study is included in Supplementary Table 2. PBMCs samples were thawed and rested for 1 h at 37 °C in a complete RPMI medium (RPMI supplemented with penicillin–streptomycin, L-Glutamine, HEPES, non-essential amino acids, 2-Mercaptoethanol, and 10% FBS). Cells were then washed, resuspended in PBS, and plated in a 96-well plate (0.5–1 × 10^6^ cells/well). Cells were then surface stained at 4 °C for 20 min with saturating concentrations of different combinations of antibodies in the presence of fixable live/dead stain (Invitrogen). Cells were then fixed and permeabilized using Foxp3/Transcription Factor Staining Kit (eBioscience) for the detection of intranuclear and intranuclear markers. The Foxp3/Transcription Factor Staining Kit was used according to the manufacturer’s instructions. Fixed cells were stained at room temperature for 30–45 min with different combinations of intranuclear and intranuclear antibodies in Foxp3 staining buffer. Samples were acquired on a BD Fortessa X20 using BD FACSDiva8.0 (BD Biosciences), and subsequent data analysis was performed using FlowJo 10 (TreeStar). The gating strategies used for flow cytometry experiments are provided in Supplementary Figs. 1 and 4.

### Production and purification of recombinant Spike

HEK-293F cells were seeded at 1 × 10^6^ cells/mL in Freestyle 293 Expression Medium (Gibco). The next day, a transfection mix was prepared (for 200 mL of cells) of 72 mg of Wuhan-hu1 Spike-Avi-His tag plasmid and 18 mg of BirA plasmid^97^ into 11 ml of Opti-MEM, alongside 2ml of PEI-Max, and left to incubate at 37°C 5%CO2 in a shaking incubator for 7 days before harvesting for purification. The supernatant was purified using 2 mM imidazole buffer (Sigma-Aldrich) buffer during binding to a His GraviTrap (Cytiva) column and 500 mM imidazole buffer for elution. The eluted protein was then concentrated with a 100KD Amicon Ultra concentrator (Millipore) and washed with PBS before quantification using a NanoDrop. Biotinylated protein was then further purified through size exclusion chromatography using an AKTA pure system with a Superdex 200 Increase 10/300 GL column (Sigma-Aldrich) to select for fractions containing trimeric Spike.

### NK cell activation assay

Cryopreserved PBMCs were thawed, washed with RPMI 1640 supplemented with 5% FBS,100U/ml penicillin/streptomycin, and 20 mM L-glutamine (Gibco, ThermoFisher), counted using Countess II Automated Cell Counter (Invitrogen, ThermoFisher) and rested for 2 h at 37 °C. Purified SARS-CoV-2 Spike protein (as described above, 5 μg/ml) or an isotype-matched control antibody (5 μg/ml) (mIgG1κ, BD Biosciences) were immobilized on 96-well flat bottom tissue culture plates overnight at 4 °C. Plates were then washed, blocked with 5% FBS (Gibco, ThermoFisher) in RPMI 1640 supplemented as above for 30 minutes. After 30-minute incubation, the blocking medium was removed, and PBMC cells from a single study donor or internal control (non-study donor used to monitor variability between experimental batches) were added at a concentration of 0.5 × 10^6^ cells/well in RPMI 1640 supplemented as above. PBMCs were collected from participants at baseline and after one, two, or three doses of vaccine. Heat-inactivated pre- or post-vaccination matched serum (autologous serum from the same study participant), pooled serum (post-second dose serum derived from PLWH with prior SAR-CoV-2 infection and had S1 IgG binding titers (> 100μg/ml)), or pre-pandemic serum (collected before 2018) were added together with anti-CD107α APC-H7 antibody (BD Biosciences, Catalog # 561343, dilution 1 in 200) and incubated for 6h at 37 °C. GolgiStop (containing Monensin, 2 μmol/l) and GolgiPlug (containing brefeldin A, 10 μg/ml) (BD Biosciences) were added for the final 5 h of culture. After stimulation, cells were surface stained with different combinations of surface antibodies in the presence of fixable live/dead stain (Invitrogen Catalog # L34957, dilution 1 in 300). Cells were then fixed and permeabilized (CytoFix/CytoPerm; BD Biosciences), followed by intracellular cytokine staining with IFN-γ BV421 (BD Biosciences, Catalog # 562988, dilution 1 in 50) and TNF-α BV711 (BioLegend, Catalog # 554512, dilution 1 in 50). Samples were acquired on a BD Fortessa X20 using BD FACSDiva8.0 (BD Biosciences), and data were analyzed using FlowJo 10 (TreeStar). A complete list of antibodies used in the NK cell activation assay is listed in Supplementary Table 3.

### Spike-specific B cell flow cytometric analysis

One μg of biotinylated Spike was incubated with either streptavidin-conjugated allophycocyanin (APC) (Agilent, Santa Clara, California, USA) or phycoerythrin (PE) (Agilent) to generate fluorochrome-linked biotinylated tetramers as described in^5^. Cryopreserved PBMCs were thawed in PBS and cells were stained with a panel of phenotyping antibodies and biotinylated tetramers (see Supplementary Table 4). Stained cells were then washed with PBS and fixed in Cytofix/Cytoperm (BD Biosciences). Samples were acquired on a BD Fortessa X20 using BD FACSDiva8.0 (BD Biosciences), and subsequent data analysis was performed using FlowJo 10 (TreeStar). Spike-specific memory B cells were defined as CD19^+^ CD20^+^ CD38^low^/^−^ IgD^−^ MBCs (excluding CD21^+^ CD27^−^ cells) Spike-PE^+^ Spike-APC^+^ gate, as defined in^5^.

### Ex-vivo IFN-γ ELISpot assay

IFN-γ ELISpot assays were performed to measure Spike-specific T-cell responses^64^. ELISPOT plates (S5EJ044I10; Merck Millipore, Darmstadt, Germany) pre-wetted with 30 µl of 70% ethanol for a maximum of 2 min, washed with sterile PBS and coated overnight at 4 °C with anti-IFN-γ antibody (10 µg/ml in PBS; clone 1-D1K; Mabtech, Nacka Strand, Sweden). Following overnight incubation, plates were washed with PBS and blocked with R10 (RPMI supplemented with penicillin–streptomycin, L-Glutamine, and 10% FBS) for a minimum of 2 h at 37 °C. The cells were then plated at 2 × 10^5^ cells/well and cultured with overlapping peptide pools at 2 μg/ml or PHA (Sigma Aldrich, St Louis, MO) at 5 µg/ml as a positive control for 16–18 h at 37 °C. Plates were washed four times with 0.05% Tween/PBS (Sigma Aldrich) followed by two washes with PBS and then incubated for 2 h at room temperature with biotinylated anti-IFN-γ (1 μg/ml; clone mAb-7B6-1; Mabtech). Next, plates were washed and incubated with alkaline phosphatase-conjugated streptavidin (Mabtech) at 1:1000 dilution for 1 h. After six further washes, plates were developed using VECTASTAIN® Elite ABC-HRP according to the manufacturer’s instructions (Mabtech). All assays were performed in duplicate. Spots were counted using an automated ELISpot Reader System.

### Overlapping peptide pools

For the detection of Spike-specific or CMV-specific T cell responses, purified cryopreserved PBMCs were stimulated with the following peptide pools: (1) SARS-CoV-2 Spike (Wuhan Hu-1) PepTivator® protein pools (Miltenyi Biotec, Gladbach, Germany). (2) CMV; peptide pools of the pp65 protein of human cytomegalovirus (CMV) (Miltenyi Biotec).

### Standardized ELISA for measurement of CMV-specific IgG levels in plasma

CMV-specific IgG titers were measured using the Abcam Anti-Cytomegalovirus (CMV) IgG Human ELISA kit following the manufacturer’s instructions. Assays were run in duplicate, and the mean values per participant were reported in International Units (IU) per ml.

### Measurement of soluble biomarkers

Cryopreserved plasma samples collected from whole blood were used to measure soluble biomarkers. The levels of plasma soluble biomarkers were measured using the Luminex multiplex bead assay on a BioPlex-100 instrument according to the manufacturer’s protocol and recommended dilutions (Luminex technology, Hertogenbosch, Netherlands). Biomarker levels (pg/mL) were calculated from a standard curve using standards of known concentration. All plasma samples were measured on the same machine, used on their first thaw and measured in duplicate, with a mean value taken from the two measurements. The full list of biomarkers analyzed is listed in Supplementary Table 5.

### Quantification and statistical analysis

Prism 8 (GraphPad Software) was used for statistical analysis as follows: the Mann–Whitney U-test was used for single comparisons of independent groups, and the Wilcoxon-signed rank test was used to compare two paired groups. In addition, the non-parametric Spearman test was used for correlation analysis and unadjusted *p* values are displayed in the graphs. Adjusted *p* values after Benjamini–Hochberg correction are presented in the figure legends (for significant correlations only).The statistical significances are indicated in the figure legends (*p < 0.05, **p < 0.01, ***p < 0.001, and ****p < 0.0001), and all tests were two-tailed.

### Supplementary Information

Supplementary Information.

## Data Availability

All the data presented in this study are available in the published article and summarized in the corresponding tables, figures and supplemental materials. Further requests can be made to the corresponding author.
