# Supplementary material for: Natural killer cell responses during SARS-CoV-2 infection and vaccination in people living with HIV-1
Source: Sci Rep. 2023 Nov 3;13:18994. doi: 10.1038/s41598-023-45412-9 (PMC10624865; doi:10.1038/s41598-023-45412-9)
Supplement: Supplementary file 1 — Supplementary Information. [file 41598_2023_45412_MOESM1_ESM.docx]

**Supplementary information:**

**Natural killer cell responses during SARS-CoV-2 infection and vaccination in people living with HIV-1**

Aljawharah Alrubayyi^1,2^, Emma Touizer^2^, Dan Hameiri-Bowen^1^, Bethany Charlton^1^, Ester Gea- Mallorquí^1^, Noshin Hussain^2^, Kelly Da Costa^2^, Rosemarie Ford^2^, Chloe Rees-Spear^2^, Thomas A Fox^2^, Ian Williams^3^, Laura Waters^3^, Tristan J Barber ^4,6^, Fiona Burns ^4,5^, Sabine Kinloch^2,5^, Emma Morris^2^, Sarah Rowland-Jones^1^, Laura E McCoy^2^, Dimitra Peppa^2,3,5^

**Supplementary fig. 1:** *Ex-vivo* analysis of NK cell subsets and soluble markers. Related to Figure 1.

**Supplementary fig. 2:** The associations between adaptive NK cells and vaccine-induced antibody and T cell responses. Related to Figure 2.

**Supplementary fig. 3:** Analysis of antibody-dependent NK cell responses. Related to Figure 3.

**Supplementary fig. 4:** Examples of gating strategies used in antibody-dependent NK cell responses. Related to Figure 4.

Supplementary Table 1. Cohort Demographics and Clinical Characteristics.

Supplementary Table 2. List of antibodies used in NK cell phenotypic assay.

Supplementary Table 3. List of antibodies used in NK cell activation assay.

**Supplementary Table 4.** List of antibodies used in spike-specific B cell phenotypic assay.

**Supplementary Table 5.** List of biomarkers.

**Supplementary fig.1: *Ex-vivo* analysis of NK cell subsets and soluble markers. Related to Fig.1.**

**(a)** Example of gating strategy used to identify NK cell subsets. Cells were gated on a single lymphocyte population, followed by live cells gating and excluding dead, CD4, CD19, and CD14 cells. Following gating on live CD3^-^ T cells, cells were gated on CD56^+^/^-^ and CD7^+^/^-^. Total NK cells were then identified by gating in CD16^+^/^-^ and CD56^+^/^-^. (**b)** Summary analysis of the frequency of CD38^+^NK cells in SARS-CoV-2 negative (green) and SARS-CoV-2-positive (red) PLWH. Filled red dots: mild (non-hospitalized cases); Opened circle: hospitalized cases. Frequencies of **(c)** GranzB^+^CD56^bright^, (**d)** NKG2A^+^, and (**e)** NKG2C^+^CD57^+^NK cells in the two study groups. The percentage of **(f)** CD16^+^ and **(g)** CD16 MFI in total NK cells in the two study groups. Correlation between FcεRIγ^+^CD57^+^ NK cells with (**h)** S1 IgG-specific titers and (**i)** N-specific IgG titers in convalescent PLWH. **(j)** Correlation between FcεRIγ^+^CD57^+^ NK cells and HCMV IgG titers in SARS-CoV-2 naïve PLWH. The levels of (**k)** HCMV IgG titers and (**l)** plasma IP10 in both study groups. Significance determined by two-tailed Mann−Whitney U test [(b), (c), (d), (e), (f), (g), (k), (l)]. Non-parametric Spearman test (two-tailed) was used for correlation analysis (unadjusted p value displayed) [(h), (i), (j)]. Data are represented as geometric mean ± SEM [(b), (c), (d), (e), (f), (g), (k), (l)].

**Supplementary fig. 1: *Ex-vivo* analysis of NK cell subsets and soluble markers. Related to Fig.1.**

**Supplementary fig. 2: The associations between adaptive NK cells and vaccine-induced antibody and T cell responses in PLWH. Related to Fig.2.**

**(a)** Longitudinal analysis of the frequency of NKG2C^+^CD57^+^CD56^dim^ NK cells in PLWH, with or without prior SARS-CoV-2 infection, at baseline (pre-vaccine) and following first, second, and third dose of vaccine. Correlation between FcεRIγ^+^CD57^+^ CD56^dim^ NK cells and S1 IgG titers after **(b)** first dose and **(c)** third dose of vaccine in PLWH, with or without prior SARS-CoV-2 infection. Correlations between FcεRIγ^+^CD57^+^CD56^dim^ NK cells and SARS-CoV-2 naturalization titers (ID_50_) after **(d)** first dose and **(e)** third dose of vaccine. Correlations between the magnitude of Spike-specific T cell responses and FcεRIγ^+^CD57^+^ NK cell frequencies after **(f)** first dose, **(g)** second dose, and **(h)** third dose of vaccine. Significance determined by two-tailed Wilcoxon-signed rank test [(a)]. Non-parametric Spearman test (two-tailed) was used for correlation analysis [(b, prior SARS-CoV-2 adjusted p value = 0.0473), (c), (d), (e), (f, prior SARS-CoV-2 adjusted p value = 0.0478), (g), (h)].

**Supplementary fig. 2: The associations between adaptive NK cells and vaccine-induced antibody and T cell responses in PLWH. Related to Fig.2.**

**Supplementary fig. 3: Analysis of antibody-dependent NK cell responses. Related to Fig.3.**

**(a)** The percentage of IFN-γ^+^ NK cells from an HIV-positive individual following 6-h stimulation with 2.5, 5, and 10 μg/ml Spike protein and different concentrations of pre-pandemic (a negative control) and pooled (a positive control) serum (top panel). The percentage of IFN-γ^+^, CD107a^+^, and TNF-α^+^ NK cells following stimulation with 5 μg/ml of Spike protein and different concentrations of pre-pandemic and pooled serum (bottom panel; serial dilutions: 0%, 0.65%, 1.25%, 2.5%, 5%, 10%). The percentage of TNF-α^+^CD56^dim^ NK cells in **(b)** cross-sectional **(c)** and longitudinal analyses in PLWH with or without prior infection. **(d)** The percentage of IFN-γ^+^CD56^dim^ NK cells in response to stimulation with pre-pandemic serum in SARS-CoV-2 naïve donors at baseline (pre-vaccine). **(e)** Paired analysis showing the percentage of IFN-γ^+^CD56^dim^ NK cells at baseline and after two doses in SARS-CoV-2 naïve individuals with pre-existing responses (red) or without pre-existing responses (green). **(f)** Pie charts representing the proportion of CD56^bright^, CD57^-^, CD57^+^FcεRIγ^+/^, CD57^+^FcεRIγ^-^ and CD57^+^NKG2C^+/-^ IFN-γ^+^ NK cells in two SARS-CoV-2 naïve donors with pre-existing responses. Significance determined by two-tailed Mann−Whitney U test [(b)], or Wilcoxon-signed rank test [(c)]; *p < 0.05, **p < 0.01. Data are represented as geometric mean ± SEM [(b)].

**Supplementary fig. 3: Analysis of antibody-dependent NK cell responses. Related to Fig.3.**

**Supplementary fig. 4: Examples of gating strategies used in antibody-dependent NK cell responses. Related to Figure 4.**

**(a)** Representative flow plots showing the expression of IFN-γ within CD56^bright^/CD56^dim^, CD57^+^/^-^, CD57^+^FcεRIγ^+^/ ^-^, and CD57^+^NKG2C^-^/^+^ cells. **(b)** Representative examples showing the expression of CD107a within different NK cell subsets.

**Supplementary fig. 4: Examples of gating strategies used in antibody-dependent NK cell responses. Related to Fig.4.**

^1^ Severity of COVID-19 was classified according to the WHO (World Health Organisation) clinical progression scale

^2^ HCMV seropositivity was determined by CMV IgG seropositivity (enzyme-linked immunosorbent assay “ELISA”) or CMV-specific T cells (IFN-γ ELISpot assay).

^3^ BAME= Black, Asian and minority ethnic.

^4^ Participants had organ transplantation, including renal or liver transplantation.

^5^ Hepatitis B virus (HBV) co-infection.

Supplementary Table 1. Cohort Demographics and Clinical Characteristics.

Supplementary Table 2. List of antibodies used in NK cell phenotypic assay.

| Antigen/mAb | Fluorochrome | Supplier | Identifier | Clone | Dilution |
| --- | --- | --- | --- | --- | --- |
| CD16 | BB700 | BD Biosciences | Cat # 746199 | Clone # 3G8 | 0.25 in 50μl |
| FcεRI | FITC | Milli-Mark | Cat # FCABS400F | Polyclonal | 0.25 in 50μl |
| CD57 | BV421 | BD Biosciences | Cat # 563896 | Clone # NK-1 | 0.25 in 50μl |
| CD4 | BV510 | BioLegend | Cat # 300546 | Clone # RPA-T4 | 0.25 in 50μl |
| CD14 | BV510 | BioLegend | Cat # 301842 | Clone # M5E2 | 0.25 in 50μl |
| CD19 | BV510 | BioLegend | Cat # 302242 | Clone # HIB19 | 0.25 in 50μl |
| Live Dead stain | Aqua | Life Technologies | Cat # L34957 | N/A | 1 in 800μl |
| CD56 | BV605 | BD Biosciences | Cat # 562780 | Clone # NCAM16.2 | 1 in 50μl |
| CD3 | BV650 | BioLegend | Cat # 317324 | Clone # OKT3 | 0.5 in 50μl |
| CD2 | BV711 | BioLegend | Cat # 300231 | Clone # RPA-2.10 | 0.5 in 50μl |
| CD38 | BV785 | BioLegend | Cat # 303530 | Clone # HIT2 | 1 in 50μl |
| KIR2DL1/KIR2DS5 | APC | R&D systems | Cat # FAB1844A-100 | Clone # 143211 | 1 in 50μl |
| KIR3DL2/ CD158K | APC | R&D systems | Cat # FAB2878A-100 | Clone # 539304 | 1 in 50μl |
| KIR3DL1/ CD158e | APC | Miltenyi Biotec | Cat # 130-092-474 | Clone # DX9 | 1 in 50μl |
| CD158b1/b2 | APC | Beckman Coulter | Cat # A22333 | Clone # GL183 | 1 in 50μl |
| Granzyme B | Alexa Fluor®700 | BD Biosciences | Cat # 560213 | Clone # GB11 | 0.5 in 50μl |
| Siglec-7 | APC/Fire-750 | BioLegend | Cat # 339207 | Clone # 434 | 0.5 in 50μl |
| NKG2C/  CD159c | PE | R&D systems | Cat # FAB138P-100 | Clone # 134591 | 5 in 50μl |
| NKG2A/  CD159a | PE-Cy-7 | Beckman Coulter | Cat # B10246 | Clone # Z199 | 0.5 in 50μl |
| PLZF | PE-CF594 | BD Biosciences | Cat # 565738 | Clone # R17-809 | 0.5 in 50μl |
| CD7 | PE/Cyanine5 | BioLegend | Cat # 343110 | Clone # CD7-6B7 | 0.25 in 50μl |

Supplementary Table 3. List of antibodies used in NK cell activation assay.

| Antigen/mAb | Fluorochrome | Supplier | Identifier | Clone | Dilution |
| --- | --- | --- | --- | --- | --- |
| CD16 | BB700 | BD Biosciences | Cat # 746199 | Clone # 3G8 | 0.25 in 50μl |
| FcεRI | FITC | Milli-Mark | Cat # FCABS400F | Polyclonal | 0.25 in 50μl |
| IFN-γ | BV421 | BD Biosciences | Cat # 562988 | Clone # B27 | 1 in 50μl |
| CD4 | BV510 | BioLegend | Cat # 300546 | Clone # RPA-T4 | 0.25 in 50μl |
| CD14 | BV510 | BioLegend | Cat # 301842 | Clone # M5E2 | 0.25 in 50μl |
| CD19 | BV510 | BioLegend | Cat # 302242 | Clone # HIB19 | 0.25 in 50μl |
| Live Dead stain | Aqua | Life Technologies | Cat # L34957 | N/A | 1 in 300μl |
| CD56 | BV605 | BD Biosciences | Cat # 562780 | Clone # NCAM16.2 | 1 in 50μl |
| CD3 | BV650 | BioLegend | Cat # 317324 | Clone # OKT3 | 0.5 in 50μl |
| TNF-α | BV711 | BioLegend | Cat # 502940 | Clone #  MAb11 | 1 in 50μl |
| CD38 | BV785 | BioLegend | Cat # 303530 | Clone # HIT2 | 1 in 50μl |
| KIR2DL1/KIR2DS5 | APC | R&D systems | Cat # FAB1844A-100 | Clone # 143211 | 1 in 50μl |
| KIR3DL2/ CD158K | APC | R&D systems | Cat # FAB2878A-100 | Clone # 539304 | 1 in 50μl |
| KIR3DL1/ CD158e | APC | Miltenyi Biotec | Cat # 130-092-474 | Clone # DX9 | 1 in 50μl |
| CD158b1/b2 | APC | Beckman Coulter | Cat # A22333 | Clone # GL183 | 1 in 50μl |
| Granzyme B | Alexa Fluor®700 | BD Biosciences | Cat # 560213 | Clone # GB11 | 0.5 in 50μl |
| CD107a | APC/H-7 | BD Biosciences | Cat # 561343 | Clone # H4A3 | 1 in 200μl |
| NKG2C/  CD159c | PE | R&D systems | Cat # FAB138P-100 | Clone # 134591 | 5 in 50μl |
| NKG2A/  CD159a | PE-Cy-7 | Beckman Coulter | Cat # B10246 | Clone # Z199 | 0.5 in 50μl |
| CD57 | PE/Dazzle 594 | BioLegend | Cat # 359620 | Clone # HNK-1 | 0.25 in 50μl |

**Supplementary Table 4.** List of antibodies used in spike-specific B cell phenotypic assay.

| Antigen/mAb | Fluorochrome | Supplier | Identifier | Clone | Dilution |
| --- | --- | --- | --- | --- | --- |
| IgG | FITC | BD Biosciences | Cat # 560952 | Clone # G18-145 | 2.5 in 50μl |
| CD19 | BV786 | BD Biosciences | Cat # 740968 | Clone # HIB19 | 1 in 50μl |
| CD27 | BUV395 | BD Biosciences | Cat # 563815 | Clone # L128 | in 50μl |
| IgD | PE-Cy™7 | BD Biosciences | Cat # 561314 | Clone # IA6-2 | 0.5 in 50μl |
| IgM | APC/Cyanine7 | BioLegend | Cat # 314520 | Clone #  MHM-88 | 0.5 in 50μl |
| CD20 | Alexa Fluor® 700 | BD Biosciences | Cat # 560631 | Clone # 2H7 | 0.5 in 50μl |
| CD21 | BV711 | BD Biosciences | Cat # 563163 | Clone #  B-ly4 | 0.5 in 50μl |
| CD38 | PE-CF594 | BD Biosciences | Cat # 562288 | Clone # HIT2 | 0.25 in 50μl |
| CD3 | BV510 | BioLegend | Cat # 317332 | Clone # OKT3 | 0.25 in 50μl |
| CD14 | BV510 | BioLegend | Cat # 301842 | Clone #  M5E2 | 0.25 in 50μl |
| Streptavidin | BV421 | BioLegend | Cat # 405226 | N/A | 1 in 50μl |
| Streptavidin | PE | Agilent | Cat # PJRS25-1 | N/A | 1 in 50μl |
| Streptavidin | APC | Agilent | Cat # PJ25S | N/A | 1 in 50μl |

**Supplementary Table 5. List of biomarkers.**

| **Biomarker** | **Bead Region** | **Dilution** | **Standard curve (pg/mL)** |
| --- | --- | --- | --- |
| VEGF | 26 | 1:2 | 0.89-217 |
| IL-6 | 13 | 1:2 | 0.49-118 |
| sCD14 | 29 | 1:200 | 21-5103 |
| CRP | 62 | 1:200 | 10.37-2520 |
| sCCL5/RANTES | 36 | 1:50 | 2.25-547 |
| D-Dimer | 43 | 1:4000 | 0.03-7.6 |
| Angiopoietin-1 | 65 | 1:2 | 12.63-3069 |
| sCCL2 | 25 | 1:2 | 3.41-828 |
| sCD25 | 47 | 1:2 | 2.7-657 |
| sCD40-L | 74 | 1:2 | 74.6-18129 |
| CXCL8/IL-8 | 18 | 1:2 | 0.47-114 |
| Fas | 73 | 1:2 | 14.58-3544 |
| IL-12p70 | 43 | 1:2 | 3.5-851 |
| TNF-R1 | 66 | 1:2 | 22.15-5382 |
| Angiopoietin-2 | 26 | 1:2 | 25.77-6263 |
| sCD163 | 28 | 1:2 | 519.56-126252 |
| sCD27 | 67 | 1:2 | 17.06-4146 |
| IP-10 | 21 | 1:2 | 0.28-69 |
| Fas-L | 39 | 1:2 | 1.78-433 |
| IFN-y | 29 | 1:2 | 4.93-1197 |
| IL-10 | 22 | 1:2 | 0.37-89 |
